# Risk factors of coronary artery disease in metabolic-associated fatty liver disease: a single center cross-sectional study

**DOI:** 10.3389/fcvm.2026.1714942

**Published:** 2026-02-27

**Authors:** Rui Jin, Xumin Wang, Tao Xu, FenFang Zhang, Lei Li

**Affiliations:** 1 Department of Gastroenterology, Beijing Jishuitan Hospital, Capital Medical University, Beijing, China; 2 Department of Cardiology, The First People’s Hospital of Yangquan, Yangquan, Shanxi, China

**Keywords:** coronary artery disease, metabolic-associated fatty liver disease, model, monocyte to high-densitylipoprotein cholesterol ratio, risk fators

## Abstract

**Introduction and objectives:**

The specific risk factors contributing to coronary artery disease (CAD) in individuals with metabolic-associated fatty liver disease (MAFLD) have not been comprehensively examined. Given the critical role of inflammation in the pathogenesis of atherosclerotic cardiovascular disease (ASCVD), the monocyte to high-density lipoprotein cholesterol ratio (MHR) has emerged as a novel and significant inflammatory biomarker linked to CAD. Therefore, the primary objective of this study was to identify the risk factors associated with CAD in the MAFLD population, to analyze and compare MHR levels in MAFLD patients with and without concurrent CAD, and to evaluate the diagnostic and predictive value of MHR for CAD incidence within this high-risk cohort.

**Materials and methods:**

In total, 251 patients with MAFLD, comprising 151 individuals with CAD and 100 without CAD, were included in the study conducted at the First People's Hospital of Yangquan. The diagnosis of CAD was established through coronary angiography. Biochemical indices were collected and subjected to logistic regression analysis to identify markers that exhibited differential expression between MAFLD patients with CAD and those without CAD. These markers were subsequently integrated into a diagnostic model. The predictive efficacy of this model was evaluated using Decision Curve Analysis (DCA). Furthermore, the relationship between the MHR and other markers was examined using Spearman's correlation analysis.

**Results:**

Gender, age, white blood cell count (WBC), total cholesterol (TC), and the MHR were identified as significant risk factors for CAD in patients with MAFLD. Based on the area under the curve (AUC) value, the diagnostic model incorporating these five risk factors demonstrated robust diagnostic performance, with an AUC of 0.809. The DCA further validated its diagnostic effectiveness. Additionally, MHR was found to possess substantial diagnostic value for the occurrence of CAD events in MAFLD patients, with an AUC of 0.701. Further analysis of MHR quartiles revealed that patients in the highest quartile exhibited a significantly elevated risk of CAD compared to those in the other three quartiles. Moreover, significant correlations were observed between MHR and body mass index, WBC, uric acid, creatinine and triglycerides.

**Conclusion:**

The findings confirmed the importance of gender, age, WBC, TC, and MHR in predicting the occurrence of CAD in MAFLD populations, with MHR showing higher predictive value.

## Introduction

Metabolic-associated fatty liver disease (MAFLD), previously referred to as non-alcoholic fatty liver disease, is a chronic hepatic condition resulting from systemic metabolic dysfunctions (1). Globally, the prevalence of MAFLD ranges from 33% to 39.22%, with the highest incidence observed in Europe and Asia, followed by North America (2–4). Driven by urbanization and demographic aging, the prevalence of MAFLD in China is projected to rise significantly, from 243.66 million cases in 2016 to 314.58 million by 2030 (5). Currently, MAFLD impacts nearly one-quarter of the adult population, posing substantial economic and health challenges on a global scale (6, 7). In individuals diagnosed with MAFLD, liver disease is not the primary cause of mortality; rather, cardiovascular events and complications are the leading causes of death (8). This is understandable, given that hepatic lipid accumulation is frequently linked with insulin resistance, obesity, and metabolic syndrome, all of which are strongly correlated with increased cardiovascular risk. An analysis of a dataset comprising 19,617 non-pregnant U.S. adults aged 20 years and older revealed a significantly elevated risk of atherosclerotic cardiovascular disease (ASCVD) in patients with MAFLD and NAFLD (10.3%–11.8%) compared to those without these conditions (6.2%–7.4%) (9). A study conducted by Lee et al. evaluated the risk of incident cardiovascular disease utilizing a Korean health screening database comprising 9,584,399 participants, with a median follow-up duration of 10.1 years. Participants were categorized into groups based on the presence of Neither-fatty liver disease (Neither-FLD), NAFLD only, MAFLD only, or both FLD types. The findings indicated that both NAFLD and MAFLD were significantly associated with an elevated risk of cardiovascular disease events. Using the Neither-FLD group as a reference, the multivariable-adjusted hazard ratios [95% confidence intervals (CI)] for cardiovascular disease events were 1.09 (1.03–1.15) for the NAFLD-only group, 1.43 (1.41–1.45) for the MAFLD-only group, and 1.56 (1.54–1.58) for the group with both FLD types (4). These results suggest that transitioning from NAFLD to MAFLD criteria may enhance the identification of individuals with metabolically complex fatty liver disease who are at an increased risk of cardiovascular disease. Currently, numerous studies have confirmed that the risk of cardiovascular disease events is significantly elevated in patients with MAFLD or NAFLD. Existing research and guidelines indicate that the absolute number of patients identified under the MAFLD criteria exceeds those identified under the NAFLD criteria, suggesting that employing the MAFLD definition may facilitate earlier treatment for a greater number of patients (4, 9–12). Nevertheless, limited research has delved into the specific risk factors for cardiovascular events in patients with either MAFLD or NAFLD. Considering that patients with MAFLD experience a more rapid deterioration in ASCVD risk compared to those with NAFLD, and that the MAFLD diagnosis is more effective in identifying high-risk individuals, this study aims to further investigate the risk factors associated with coronary artery disease (CAD) in MAFLD patients. The goal is to reduce the incidence of CAD and mitigate the growing socioeconomic burden. Additionally, numerous studies have demonstrated that inflammation plays a crucial role in the onset and progression of ASCVD. The monocyte to high-density lipoprotein cholesterol ratio (MHR) has emerged as a novel biomarker of inflammation, and its established association with CAD is well-documented (13–15). Consequently, this study aimed to compare the levels of MHR between two groups of patients with MAFLD, specifically those with and without CAD. Additionally, the study sought to evaluate the diagnostic predictive value of varying MHR levels for the incidence of CAD in this patient population.

## Methods

### Study design and the subjects

#### Data sources

**Figure 1:**
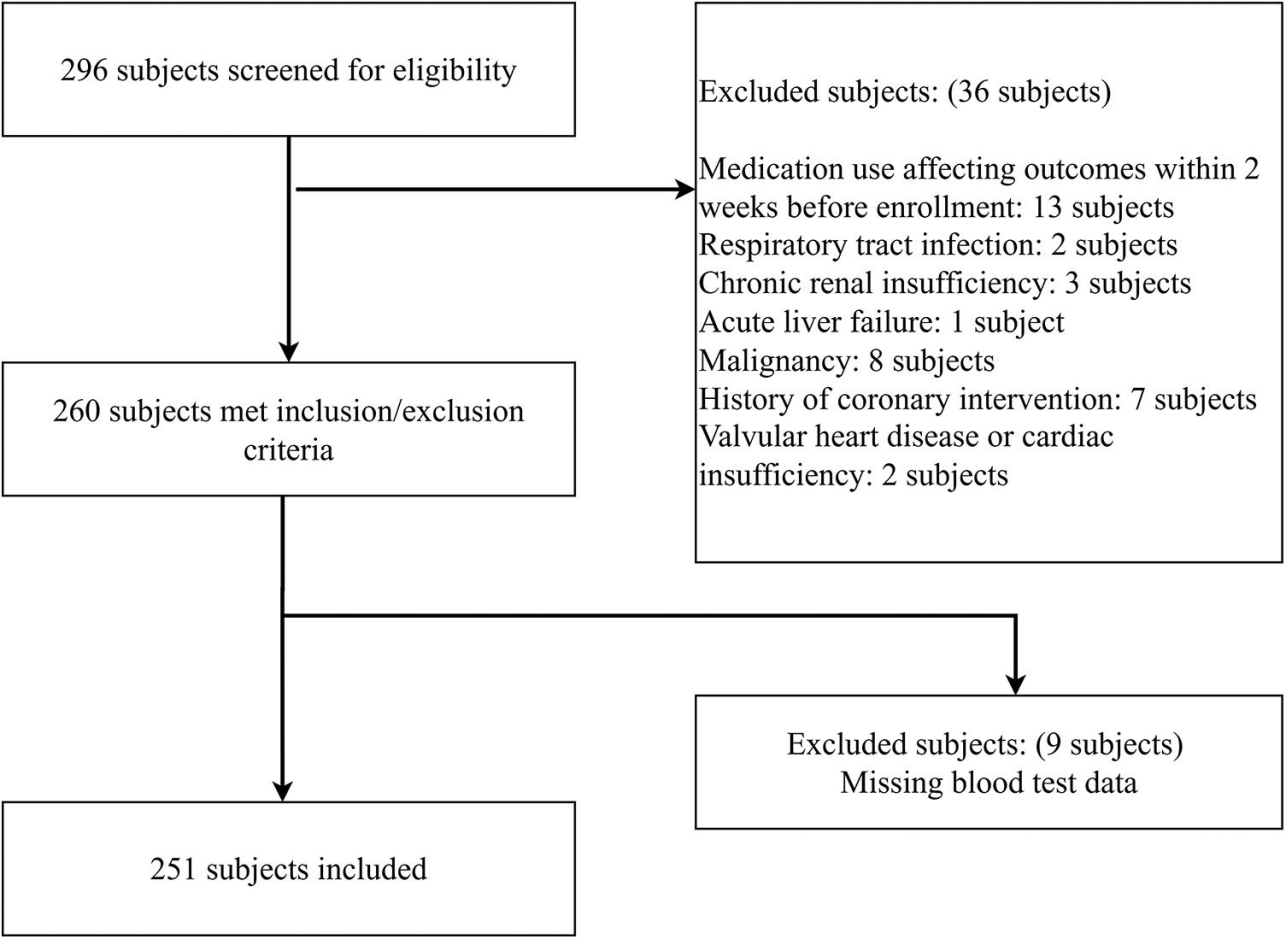
Flow diagram of participant enrollment and exclusion.

This project is a cross-sectional study. A total of 296 subjects were screened for study eligibility at Yangquan First People's Hospital between January 2022 and December 2024, with 45 excluded for failing to meet the predefined inclusion and exclusion criteria. The complete participant recruitment flow is depicted in Figure 1, which details the numbers of screened, excluded and finally enrolled subjects. Following the exclusion of ineligible individuals, 251 patients with MAFLD were included in the final analysis, among whom 151 had concomitant CAD (CAD group) and 100 had no evidence of CAD (non-CAD group). The Ethics Committee of the first People's Hospital of Yangquan has reviewed this study, with the ethics number YQDYRMYY-LLMS-2024-03.

#### Inclusion criteria

Inclusion criteria for patients with MAFLD are as follows: (1) individuals must be over 18 years of age; (2) they must not have a history of excessive alcohol consumption or other known factors that could cause liver damage. Additionally, more than 5% of liver cells must exhibit initial signs of steatosis, as determined by a physician through liver ultrasound examination. Furthermore, patients must meet at least one of the following three conditions: (1) being classified as overweight or obese; (2) having a diagnosis of type 2 diabetes; or (3) exhibiting at least two metabolic risk factors, which include: (a) a waist circumference of 90 cm or more for men and 80 cm or more for women; (b) blood pressure equal to or exceeding 130/85 mmHg, or the requirement for antihypertensive medication; (c) plasma triglyceride levels at or above 150 mg/dL, or the need for lipid-lowering medication; (d) low plasma HDL-C levels. CAD was diagnosed by experienced clinicians using coronary angiography, with the Judkins method employed to document angiographic findings. The GENSINI score was utilized to assess the degree of coronary stenosis, which is defined as a lumen diameter stenosis of ≥50% in any major coronary artery or significant branches.

#### Exclusion criteria

A patient should be excluded from the study if any of the following three criteria are met: (1) the use of medications that could influence the study's outcomes, including glucocorticoids, anti-inflammatory drugs, thyroid hormones, statins, or triglyceride-lowering agents, within the preceding 2 weeks; (2) the presence of acute or chronic infections affecting the respiratory, urinary, or digestive systems within the past 2 weeks, particularly if accompanied by severe hepatic or renal dysfunction, immune disorders, malignancies, hematological conditions, among others; (3) a history of coronary interventions such as angioplasty or coronary artery bypass grafting, or other cardiac conditions including rheumatic heart disease, valvular heart disease, cardiac syndrome X, severe congenital heart defects, cardiomyopathy, or significant heart failure.

### Data collection and measures

The following subject variables were included in the dataset: sex, age, body mass index (BMI), smoking and drinking history, hypertensive and diabetes mellitus history, white blood cells (WBC), red blood cells (RBC), Hemoglobin (HGB), platelets (PLT), fasting plasma glucose (FPG), hemoglobin A1c (HbA1c), alanine aminotransferase (ALT), Aspartate aminotransferase (AST), uric acid (UA), creatinine (Cr), blood urea nitrogen (BUN), total cholesterol (TC), triglyceride (TG), high-density lipoprotein cholesterol (HDL-C), low-density lipoprotein cholesterol (LDL-C).

### Research methods

#### Calculation of MHR

The MHR ratio serves as a clinical indicator for evaluating cardiovascular disease risk. It is determined by dividing the monocyte count by the concentration of HDL-C. Generally, an elevated MHR is associated with an increased risk of cardiovascular disease.

#### Statistical methods

All statistical analyses and graphical representations were conducted using R version 4.3.2, IBM SPSS Statistics version 26, and GraphPad Prism version 9.3.0. Results for normally distributed quantitative data are presented as the mean ± standard deviation (SD). To compare differences between two groups, both the parametric Student's *t*-test and the non-parametric Mann–Whitney *U* test were employed. Categorical data are expressed as *n* (%), and comparisons were made using the chi-square test. For comparisons across multiple groups, the non-parametric Kruskal–Wallis test was utilized. Logistic regression analysis was conducted to identify independent risk factors for MAFLD combined with CAG, with the odds ratio (OR) reported alongside a 95% CI. The receiver operating characteristic (ROC) curve was generated using the pROC package to evaluate the diagnostic efficacy of each index for metabolic associated MAFLD with CAD. The diagnostic performance was assessed by comparing the area under the curve (AUC) for each index. The optimal cut-off value was determined by identifying the maximum value of the Youden index, calculated as sensitivity plus specificity minus one. The decision curve analysis (DCA) was conducted to assess the predictive value of the model. Spearman correlation analysis was performed to evaluate the relationships between the MHR and other parameters. Statistical significance was determined at a *P*-value <0.05.

## Results

### Comparison of MAFLD patients baseline characteristics between CAD and non-CAD groups

The study encompassed a cohort of 251 MAFLD patients, comprising 148 males and 103 females, with a mean age of 61 years (±7). Among these, 100 patients were classified as non-CAD, while 151 were identified as having CAD. The comparison of baseline characteristics between these two cohorts revealed statistically significant differences in clinical and pathological features (*P* < 0.05), as detailed in Table 1. Notable statistical differences were observed between the groups concerning gender, age, WBC, FPG, HbA1c, Cr, TC, HDL-C, and MHR. Specifically, the CAD group exhibited significantly elevated levels of MHR, age, WBC, FPG, HbA1c, and Cr, alongside reduced levels of TC and HDL-C, compared to the non-CAD group. Furthermore, the CAD group had a higher proportion of male patients, whereas the gender ratio in the non-CAD group was more balanced.

**Table 1:** Comparison of MAFLD patients baseline characteristics between CAD and non-CAD groups.

Variable	Overall, *N* = 251^a^	Coronary artery disease		Statistics	*P*-value^b^
		Yes, *N* = 151 (60%)^a^	No, *N* = 100 (40%)^a^		
Sex				6.15	**0** **.** **013**
Male	148 (58.96%)	99 (65.56%)	49 (49.00%)		
Female	103 (41.04%)	52 (34.44%)	51 (51.00%)		
Age (years)	61.00 [55.00, 68.00]	63.00 [57.00, 70.00]	60.00 [54.00, 65.00]	9,011.00	**0** **.** **009**
BMI (kgm^2^)	26.83 [24.24, 29.38]	26.83 [24.05, 29.39]	26.89 [24.88, 29.30]	7,191.00	0.524
Smoking				1.62	0.202
Yes	78 (31.08%)	52 (34.44%)	26 (26.00%)		
No	173 (68.92%)	99 (65.56%)	74 (74.00%)		
Drinking				0.00	0.997
Yes	54 (21.51%)	33 (21.85%)	21 (21.00%)		
No	197 (78.49%)	118 (78.15%)	79 (79.00%)		
Hypertensive				0.03	0.857
Yes	191 (76.10%)	116 (76.82%)	75 (75.00%)		
No	60 (23.90%)	35 (23.18%)	25 (25.00%)		
Diabetes mellitus				1.47	0.226
Yes	131 (52.19%)	84 (55.63%)	47 (47.00%)		
No	120 (47.81%)	67 (44.37%)	53 (53.00%)		
WBC	6.40 [5.60, 7.76]	6.68 [5.70, 7.77]	6.15 [5.19, 7.44]	8,693.50	**0** **.** **042**
RBC	4.62 [4.35, 4.98]	4.61 [4.35, 4.95]	4.69 [4.33, 5.04]	7,087.50	0.412
HGB	145.00 [131.50, 154.00]	144.00 [132.00, 153.00]	146.00 [130.75, 157.25]	7,019.50	0.346
PLT	228.00 [190.00, 259.00]	221.00 [178.00, 255.00]	232.00 [198.75, 270.25]	6,606.50	0.094
FPG	7.50 [6.10, 9.58]	8.00 [6.37, 9.90]	6.71 [5.73, 8.71]	9,338.50	**0** **.** **001**
HbA1c	6.60 [5.95, 8.05]	7.10 [6.05, 8.30]	6.40 [5.80, 7.43]	9,282.50	**0** **.** **002**
ALT	24.00 [17.00, 33.00]	24.00 [17.00, 37.00]	24.00 [17.00, 30.00]	7,856.00	0.587
AST	22.00 [18.00, 32.00]	23.00 [17.00, 41.00]	22.00 [19.00, 27.00]	8,273.00	0.199
UA	308.00 [252.00, 349.50]	310.00 [262.00, 350.00]	295.50 [239.50, 347.25]	7,909.50	0.524
Cr	64.10 [51.60, 73.80]	66.70 [54.30, 76.65]	59.45 [49.95, 68.93]	9,231.50	**0** **.** **003**
BUN	5.17 [4.44, 6.28]	5.20 [4.42, 6.42]	5.12 [4.46, 5.90]	8,277.00	0.197
TC	4.13 [3.40, 4.91]	4.00 [3.29, 4.77]	4.32 [3.63, 5.41]	5,909.00	**0** **.** **004**
TG	1.69 [1.26, 2.43]	1.69 [1.25, 2.39]	1.68 [1.26, 2.57]	7,429.50	0.831
HDLC	0.92 [0.81, 1.09]	0.90 [0.80, 1.04]	0.99 [0.83, 1.13]	6,138.50	**0** **.** **012**
LDLC	2.53 [1.88, 3.19]	2.35 [1.80, 3.07]	2.65 [2.05, 3.41]	6,489.50	0.060
MHR	0.56 [0.39, 0.80]	0.65 [0.44, 6.04]	0.44 [0.36, 0.58]	10,592.50	**<0** **.** **001**

^a^ *n* (%); Median [IQR].

^b^ Pearson's Chi-squared test; Wilcoxon rank sum test.

The bold values emphasizes the *P*-value <0.05, which has statistical significance.

### The association between CAD and non-CAD in multivariable logistic regression analyses

**Figure 2:**
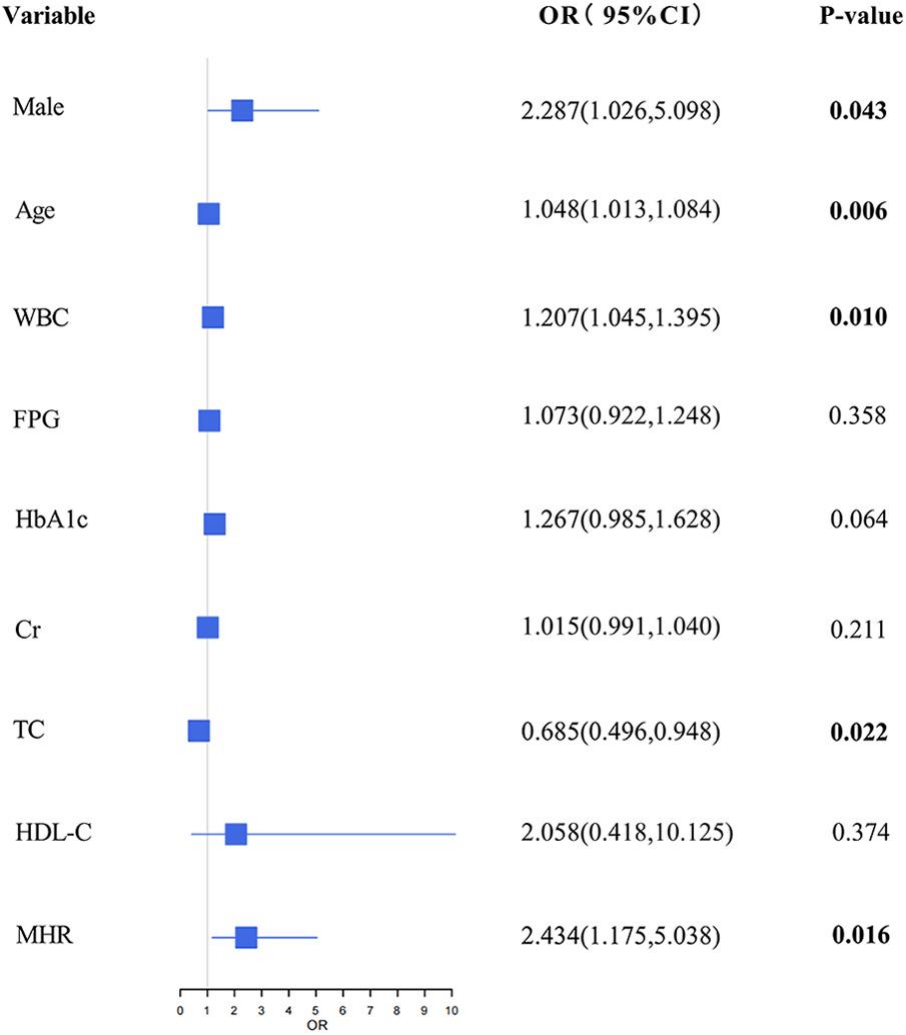
The association between CAD and non-CAD in multivariable logistic regression analyses.

Figure 2 presents the outcomes of the logistic regression analysis. This analysis identified gender, age, WBC, TC, and MHR as independent risk factors for CAD in patients with MAFLD (*P* < 0.05). There was a positive association with being male (OR = 2.287; 95% CI = 1.026–5.098; *P* = 0.043), age (OR = 1.048; 95% CI = 1.013–1.084; *P* = 0.006), WBC (OR = 1.207; 95% CI = 1.045–1.395; *P* = 0.010), and MHR (OR = 2.434; 95% CI = 1.175–5.038; *P* = 0.016). Additionally, the results indicated a significant negative correlation between TC and the risk of CAD (OR = 0.685; 95% CI = 0.496–0.948; *P* = 0.022).

### ROC curves for age, WBC, TC, MHR predicting the occurrence of CAD in MAFLD

**Figure 3:**
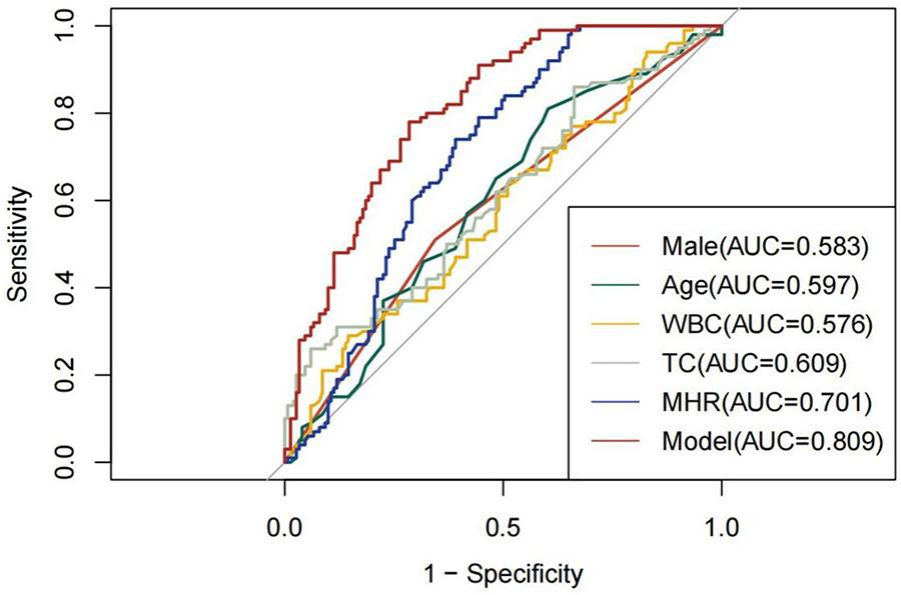
ROC curves for male, age, WBC, TC, and MHR in predicting CAD occurrence in MAFLD, along with evaluating the predictive capability of the combined model using ROC.

**Figure 4:**
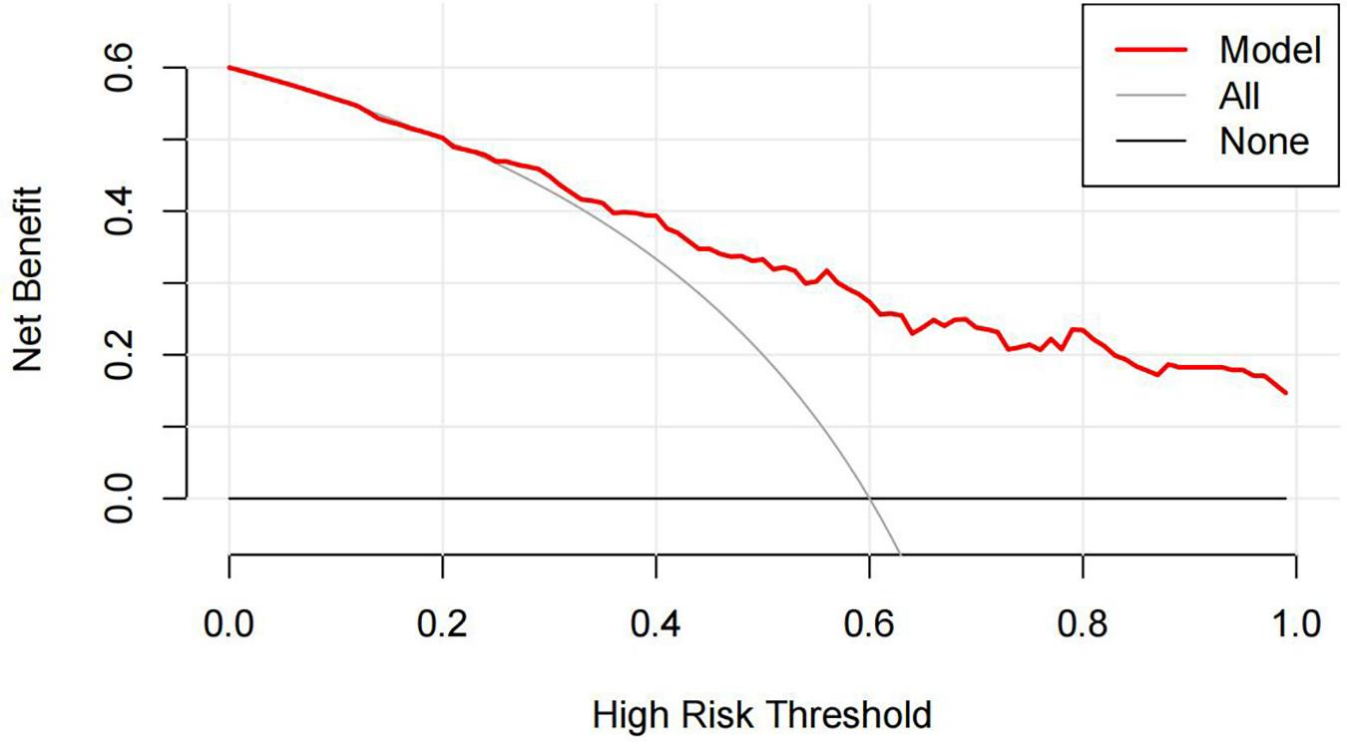
The assessment of the model decision curve analysis (DCA).

ROC curve analysis assessed the predictive ability of gender, age, WBC, TC, and MHR for CAD in MAFLD patients. Results showed: Male: AUC 0.583, 95% CI (0.521, 0.645), specificity 0.656, sensitivity 0.510, cut-off 0.500. Age: AUC 0.597, 95% CI (0.526, 0.668), specificity 0.397, sensitivity 0.810, cut-off 66.50. WBC: AUC 0.576, 95% CI (0.504, 0.648), specificity 0.854, sensitivity 0.290, cut-off 5.360. TC: AUC 0.609, 95% CI (0.537, 0.681), specificity 0.940, sensitivity 0.260, cut-off 5.390. MHR: AUC 0.701, 95% CI (0.638, 0.765), specificity 0.609, sensitivity 0.740, cut-off 0.572. Model: AUC 0.809, 95% CI (0.757, 0.861), specificity 0.715, sensitivity 0.780, cut-off 0.437. Refer to Figure 3 for details. Furthermore, DCA was conducted to evaluate the clinical utility of the model. In this analysis, “None” represented the scenario where all patients were diagnosed with MAFLD without CAD, “All” represented the scenario where all patients were diagnosed with MAFLD with CAD, and “Model” referred to the diagnostic model for identifying MAFLD with CAD. The analysis revealed that the red line, representing the model, was positioned higher than the other two curves, indicating that employing the model to diagnose MAFLD with CAD could enhance clinical benefits (see Figure 4).

### Baseline characteristics of study subjects according to the levels of MHR

Study subjects were divided into four groups (Q1–Q4) based on MHR levels. Differences in CAD, gender, BMI, diabetes mellitus, WBC, RBC, HGB, and UA were observed among these groups. Analysis indicates that patients in the highest quartile of MHR have a significantly higher risk of developing CAD compared to those in the lower three quartiles. The Q3 group had a significantly higher proportion of males and elevated BMI, WBC, RBC, HGB and UA levels compared to Q1, Q2, and Q4. The Q4 group showed the highest diabetes prevalence, while the Q3 group had the lowest (Table 2).

**Table 2:** Baseline characteristics of study subjects according to the levels of MHR.

Variable	Q1, *N* = 63 (25.1%)^a^	Q2, *N* = 64 (25.5%)^a^	Q3, *N* = 62 (24.7%)^a^	Q4, *N* = 62 (24.7%)^a^	Statistics	*P*-value^b^
CAD					36.911	**<0** **.** **001**
Yes	31 (49.21%)	26 (40.63%)	38 (61.29%)	56 (90.32%)		
No	32 (50.79%)	38 (59.38%)	24 (38.71%)	6 (9.68%)		
Sex					11.597	**0** **.** **009**
Male	29 (46.03%)	34 (53.13%)	46 (74.19%)	39 (62.90%)		
Female	34 (53.97%)	30 (46.88%)	16 (25.81%)	23 (37.10%)		
Age (years)	63.00	59.80	58.13	62.00	4.536	0.2091
BMI (kgm^2^)	26.17	26.42	28.06	28.00	11.134	**0** **.** **011**
Smoking					7.458	0.059
Yes	14 (22.22%)	17 (26.56%)	27 (43.55%)	20 (32.26%)		
No	49 (77.78%)	47 (73.44%)	35 (56.45%)	42 (67.74%)		
Drinking					1.272	0.736
Yes	14 (22.22%)	13 (20.31%)	16 (25.81%)	11 (17.74%)		
No	49 (77.78%)	51 (79.69%)	46 (74.19%)	51 (82.26%)		
Hypertensive					3.014	0.389
Yes	44 (69.84%)	47 (73.44%)	50 (80.65%)	50 (80.65%)		
No	19 (30.16%)	17 (26.56%)	12 (19.35%)	12 (19.35%)		
Diabetes mellitus					18.068	**<0** **.** **001**
Yes	37 (58.73%)	37 (57.81%)	18 (29.03%)	39 (62.90%)		
No	26 (41.27%)	27 (42.19%)	44 (70.97%)	23 (37.10%)		
WBC	5.94	6.46	8.79	7.02	55.196	**<0** **.** **001**
RBC	4.51	4.66	4.77	4.61	7.958	**0** **.** **047**
HGB	136.49	144.31	146.73	141.84	9.307	**0** **.** **026**
PLT	220.78	223.58	247.61	232.24	4.630	0.201
FPG	8.03	7.51	8.61	8.97	4.183	0.242
HbA1c	7.18	6.92	7.12	7.38	4.599	0.204
ALT	25.49	31.10	39.28	29.77	3.900	0.273
AST	27.44	28.77	46.82	32.15	4.470	0.215
UA	281.63	308.27	326.27	313.44	7.958	**0** **.** **047**
Cr	61.93	62.82	68.41	68.85	6.501	0.090
BUN	5.62	5.22	6.60	5.58	3.515	0.319
	4.35	4.30	4.32	3.93	5.836	0.120
TG	1.84	2.40	2.54	2.35	4.227	0.238
LDL-C	3.14	2.55	2.67	2.34	5.057	0.168

^a^ *n* (%); Median.

^b^ Pearson's Chi-squared test; Wilcoxon rank sum test.

The bold values emphasizes the *P*-value <0.05, which has statistical significance.

### The correlations between MHR and potential risk factors of MAFLD

**Figure 5:**
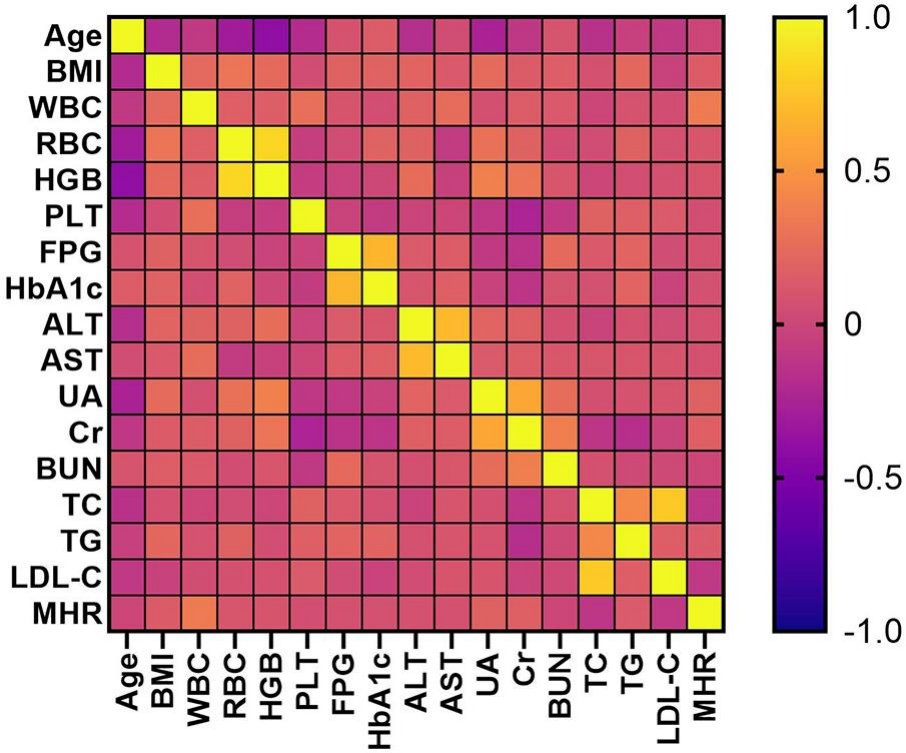
Correlation heatmaps between MHR and potential risk factors of MAFLD.

There were no significant correlations between MHR and age, BMI, RBC, HGB, PLT, FPG, HbA1c, ALT, AST, BUN, TC, or LDL-C. However, MHR showed positive correlations with BMI (*r* = 0.145, *P* = 0.022), WBC (*r* = 0.342, *P* < 0.001), UA (*r* = 0.178, *P* = 0.004), Cr (*r* = 0.171, *P* = 0.007), and TG (*r* = 0.131, *P* = 0.038). See Figure 5.

## Discussion

Recent literature has indicated that MAFLD not only elevates the risk of cardiovascular disease but also serves as an independent predictor of cardiovascular outcomes in individuals with newly diagnosed CAD (4, 9, 16, 17). A meta-analysis encompassing 34 studies conducted between 1965 and 2015, which included a total of 164,494 participants, established that NAFLD is an independent risk factor for increased rates of cardiovascular events (18). Furthermore, prospective studies have reported a positive correlation between the severity of cardiovascular disease risk and the extent of underlying lesions in NAFLD (19). While the current cohort study primarily focuses on the diagnosis of patients with NAFLD, it is evident that NAFLD independently elevates the risk of cardiovascular disease events, irrespective of traditional risk factors such as dyslipidemia, hypertension, obesity, type 2 diabetes, and insulin resistance (20). However, there has been a paucity of research examining the factors influencing CAD in the context of MAFLD. Consequently, this study seeks to address this gap by incorporating statistically significant indicators identified through univariate analysis into a logistic regression model for feature selection and filtering. The analysis revealed that gender, age, WBC, TC, and the MHR are significant risk factors for CAD in patients with MAFLD. Both CAD and MAFLD are known to be influenced by age, sex, abnormal cholesterol metabolism, and menopausal status (19). Previous studies have corroborated these findings. Research indicates that the prevalence of NAFLD is higher in men than in women (21, 22). However, after the age of 50, the prevalence becomes similar between the sexes, potentially correlating with the onset of menopause in women. This change is associated with decreased estrogen levels and increased intra-abdominal fat distribution, both of which contribute to heightened insulin resistance (23). The Prospective Multicenter Imaging Study for Evaluation of Chest Pain trial revealed significant sex-based differences in five global risk scores, including Framingham, ASCVD, Diamond and Forrester, modified Diamond and Forrester, and Diamond-Forrester and Cass. Both event risk and pretest likelihood of coronary artery disease were higher in men than in women (24). Furthermore, a study involving 665 NAFLD patients identified that advancing age, BMI, and estimated glomerular filtration rate (eGFR) were significantly correlated with coronary artery calcium scores greater than 100 (25). In a separate study, Danielsen et al. conducted coronary CT angiography on 57 patients with cirrhosis who did not have cardiovascular disease. They found that coronary calcification scores were age-dependent but were not associated with established cardiovascular risk factors, such as smoking, type 2 diabetes, hypertension, sex, or hypercholesterolemia (26). In our study, we did not observe any association between smoking, type 2 diabetes, hypertension, and coronary atherosclerosis. However, age emerged as a significant risk factor for coronary atherosclerosis in patients with MAFLD, with arteriosclerosis being particularly notable in individuals older than 66 years, serving as a critical diagnostic cutoff for predicting coronary atherosclerosis in this population.

Additionally, TC was identified as an independent predictor of CAD in MAFLD patients. Furthermore, TC levels were significantly associated with CAD in MAFLD. Traditionally, MAFLD patients are characterized by an atherogenic dyslipidemia profile, which includes elevated levels of TG, TC, LDL-C, chylomicrons, and very low-density lipoprotein, along with reduced levels of HDL-C and apolipoprotein A-I (20, 27). This dyslipidemic profile is strongly linked to adverse cardiovascular outcomes. Interestingly, our findings revealed that patients with MAFLD and CAD exhibited lower TC levels compared to those with MAFLD without CAD. Notably, our findings regarding the TC levels in patients with MAFLD complicated by CAD are inconsistent with the conventional understanding that elevated TC is a key risk factor for CAD development. This discrepancy, which was not anticipated in our initial study design, prompts us to propose potential underlying mechanisms for this phenomenon. On the one hand, patients with MAFLD are frequently complicated by dyslipidemia, a well-recognized risk factor for CAD, and thus many of them have already received lipid-lowering therapy with statins and other relevant agents prior to the onset of CAD. Such pre-emptive pharmacological intervention is likely to result in iatrogenic reduction in TC levels, thereby masking the inherent association between MAFLD-related dyslipidemia and CAD. On the other hand, when MAFLD patients progress to complicated CAD, they are mostly accompanied by aggravated metabolic disturbance, further progression of MAFLD, and systemic pathological remodeling. These comprehensive pathophysiological changes can lead to decreased *de novo* cholesterol synthesis as well as increased cholesterol consumption and loss in the body, thereby causing a physiological reduction in TC levels. This phenomenon represents a pathologically induced decline in TC, which is commonly observed in patients with moderate to advanced stages of MAFLD and CAD (28, 29). Additionally, ultrasound was used as the tool for screening and diagnosing MAFLD in this study. Its low sensitivity for mild steatosis may introduce bias in the included data, resulting in a false reduction in TC levels. Recently, international hepatology organizations have proposed “metabolic dysfunction-associated steatotic liver disease (MASLD)”, which further refines the role of metabolic dysfunction in liver diseases and emphasizes cardiovascular disease risk factors. In future studies, we will enroll patients who meet the diagnostic criteria for MASLD, compare the incidence of CAD and differences in related risk factors between MAFLD and MASLD patients, and further explore the trend of TC levels.

Beyond these considerations, the link between MAFLD, inflammation, and CAD can be further elucidated through monocytes and related inflammatory biomarkers, key mediators of arteriosclerosis. While cholesterol-induced monocyte activation is a well-established in arteriosclerosis pathogenesis, a study of 554 dyslipidemic patients and 246 normolipidemic controls found that WBC count—rather than C-reactive protein—was associated with early arteriosclerosis and its progression, identifying WBC as an independent risk factor for this condition (30). Consistent with this, our study further confirms that WBC as an independent risk factor for coronary atherosclerosis in MAFLD patients, underscoring the role of chronic low-grade inflammation in arteriosclerosis. Monocytes, in particular, are the primary source of pro-inflammatory substances in arteriosclerosis progression, and numerous studies have recognized the MHR as a novel inflammatory biomarker closely linked to cardiovascular disease. Notably, insulin resistance (IR) acts as the core link in the pathogenesis of MAFLD. On the one hand, IR promotes the release of inflammatory cytokines and chemokines, including tumor necrosis factor-α (TNF-α) and interleukin-6, among which TNF-α initiates monocyte activation. On the other hand, IR impairs lipid metabolism, leading to elevated TC and LDL-C levels, reduced HDL-C levels, and inhibited HDL-C synthesis. Meanwhile, IR damages the synthesis of endothelial nitric oxide, further compromising vascular endothelial function. In addition, increased reactive oxygen species production in the liver of MAFLD patients exacerbates endothelial oxidative damage, accelerates the progression of arteriosclerosis, and forms a vicious cycle of metabolism-inflammation-vascular injury (29, 31–33). As a comprehensive biomarker of systemic inflammation and metabolic disorders in MAFLD patients, the MHR accelerates the development and progression of CAD through two core pathways: “monocyte activation → foam cell formation → plaque progression” and “endothelial dysfunction → increased vascular permeability → inflammatory cell infiltration”. Changes in MHR values can directly reflect the risk of vascular injury in MAFLD patients, providing a potential target for early clinical intervention. Relevant studies support MHR's predictive value: Kaynak et al. identified a modest linear correlation between MHR and Synergy between percutaneous coronary intervention with Taxus and Cardiac Surgery (SYNTAX) score (*R* = 0.522, *P* < 0.001), with an MHR of 15.64 (AUC = 0.794; *P* < 0.001) effectively predicting a SYNTAX score >22 (sensitivity 81.8%, specificity 78.3%) (34). Shu et al. demonstrated that MHR is an independent predictor of CAD, with an increase in MHR significantly associated with OR for CAD (OR = 4.29, 95% CI 2.72–6.78, *P* < 0.001). The AUC for predicting CAD was 0.68, with an optimal cut-off value of 0.42 (Youden Index: 0.29) (35). Compared with classical CAD risk scores, such as the Framingham Risk Score (FRS), QRISK3), MHR has distinct pros and cons in CAD risk stratification. FRS and QRISK3 focus on traditional risk factors (age, blood pressure, etc.), enabling effective population-level macro-stratification but failing to predict subclinical inflammation-driven CAD progression (36, 37). In contrast, MHR integrates monocyte-mediated inflammation and HDL lipid metabolism, capturing pathological dimensions overlooked by traditional scores—particularly valuable in MAFLD patients, where elevated MHR links to hepatic steatosis, IR, and coronary plaque burden, correcting FRS/QRISK3's risk underestimation. Clinically, MHR is simple and low-cost for primary care screening. A study of 1,720 ACS patients undergoing PCI (June 2016–November 2017) showed that middle and highest MHR tertiles had higher primary endpoint risk vs. the lowest (adjusted HR: 1.541, 95% CI: 1.152–2.060 and 1.800, 95% CI: 1.333–2.432) over 31 months of follow-up (353 events). MHR also enhanced GRACE score predictive ability (cNRI: 0.136, *P* < 0.001; IDI: 0.006, *P* < 0.001) (38). In conclusion, MHR can serve as a valuable supplement to enhance the predictive ability of CAD risk assessment.

Utilizing logistic regression analysis to screen five risk factors, we employed ROC curves to assess the diagnostic efficacy of both individual and combined factors for concurrent CAD in the context of MAFLD. Based on the AUC value, our findings indicate that the diagnostic model incorporating five risk factors demonstrates robust diagnostic performance, with an AUC of 0.809. The DCA further corroborates its efficacy. Additionally, our analysis reveals that the MHR possesses significant diagnostic value for predicting CAD events in patients with MAFLD, as evidenced by an AUC of 0.701. Further examination of MHR quartiles indicates that individuals in the highest quartile exhibit a substantially elevated risk of CAD compared to those in the lower three quartiles. Moreover, significant correlations were observed between MHR and several clinical parameters, including BMI, WBC, UA, CR and TG. We analyzed the association between MHR levels and CAD risk by dividing the study population into four groups according to MHR quartiles. Comparative analysis of data across the four quartile groups revealed a clear dose-response relationship between MHR and CAD risk: patients in the highest MHR quartile exhibited a significantly higher risk of developing CAD compared to those in the lower three quartiles. Notably, our findings are consistent with existing evidence in the field. Specifically, studies by Sun Hui et al. and Manoochehri et al. have both corroborated that MHR levels are significantly higher in patients with acute myocardial infarction (AMI) than in healthy controls (39, 40). Furthermore, these studies demonstrated a positive correlation between MHR levels, the presence of coronary artery disease, and Gensini scores—findings that align closely with the results of our MHR quartile analysis, thereby reinforcing the reliability of our observations.

This study is subject to several limitations. Firstly, as a cross-sectional study, it precludes the establishment of causality, necessitating further prospective research to substantiate these findings. Secondly, the dataset was derived from a single-center cohort, which may limit the generalizability of the results to other regions and ethnic groups, potentially introducing data bias. Thirdly, the study relied on ultrasonography for the diagnosis of MASLD, which has limitations in identifying mild steatosis and may lead to potential diagnostic bias. Future prospective studies can incorporate indicators such as ultrasound elastography and the AST/ALT ratio to reduce diagnostic bias and further validate the conclusions of this study. Finally, the control group in this study was defined as MAFLD patients who underwent coronary angiography with no significant stenosis, which differs from the ideal study design; this may introduce potential selection bias. In subsequent studies, we will enroll two cohorts (MAFLD patients with coronary angiography showing no significant stenosis and general MAFLD patients without coronary angiography) to establish an external validation cohort, aiming to further verify the reliability of the study conclusions. Consequently, this limitation constrains a comprehensive assessment of the association between risk factors and the incidence of CAD in MAFLD patients.

## Conclusion

The findings confirmed the importance of gender, age, WBC, TC, and MHR in predicting the occurrence of CAD in MAFLD populations, with MHR showing higher predictive value.

## Data Availability

The original contributions presented in the study are included in the article/Supplementary Material, further inquiries can be directed to the corresponding authors.
